# Disaster Preparedness among Healthcare Professionals in Lebanon

**DOI:** 10.3390/ijerph21081034

**Published:** 2024-08-06

**Authors:** Yara Skaff, Mohammad Jarrah, Rabih Nasrallah, Reina Habib, Rania Sakr

**Affiliations:** 1 Department of Internal Medicine, Lebanese American University Medical Center Rizk Hospital, Beirut P.O. Box 11-3288, Lebanon; 2 Gilbert and Rose-Mary Chagoury School of Medicine, Lebanese American University, Byblos P.O. Box 36, Lebanon; 3 Department of General Surgery, Lebanese American University Medical Center Rizk Hospital, Beirut P.O. Box 11-3288, Lebanon; 4 Division of Family Medicine, Department of Internal Medicine, Lebanese American University Medical Center Rizk Hospital, Beirut P.O. Box 11-3288, Lebanon

**Keywords:** knowledge, attitude, readiness to practice, disaster medicine preparedness

## Abstract

Background: Disaster disrupts the normal functioning of a community, causing significant damages and losses. In recent years, Lebanon faced multiple disasters, including one of the largest explosions ever recorded, the Beirut Blast, in August 2020. Limited studies in the literature have examined disaster medicine preparedness among healthcare professionals (HCPs). Objective: To examine the knowledge (K), attitude (A), readiness to practice (rP), and KArP associated with disaster medicine preparedness among HCPs in Lebanon. Methods: A cross-sectional observational study was conducted in Lebanon using data from participants answering an online survey. Participants enrolled in this study were HCPs (residents and faculty), medical students, and postdoctoral research scholars. Levels of knowledge, attitude, and readiness to practice were assessed and used to examine their association with participants’ socio-demographic characteristics. Results: A total of 195 participants (average age 30.6 ± 11.4 years) were included in this study. Participants reported moderate scores of knowledge, attitude, and readiness to practice. Older participants reported better readiness to practice and a KArP score. A significant difference was observed in all categories according to gender, with men having higher scores than women. No significant difference was observed between the level of education and knowledge, attitude, and total KArP scores. Conclusions: Our study’s findings showed that age and the level of education were positively correlated with readiness to practice. Men, compared to women, had significantly higher scores in all categories. Barriers to the KArP should be identified and targeted in future studies, as disaster preparedness at the institutional level may improve outcomes in future disaster encounters.

## 1. Introduction

Disaster is defined as a serious event that disrupts the normal functioning of a community, causing losses in different areas of life and exceeding the community’s capacity to manage [1]. Whether natural or man-made, disasters cause an impairment to the economy and, most importantly, can lead to the breakdown of the healthcare system which has detrimental effects [2]. Disaster medicine has emerged as a field combining both crisis healthcare and emergency management. Physicians play a critical role in disaster medicine, handling tasks from treating injuries to managing outbreaks and prioritizing patient care, and their indispensable role is paramount in reducing the impact of disasters on communities. As disasters strike unexpectedly, it is imperative for all healthcare practitioners, to possess a fundamental understanding of disaster medicine and management to effectively respond to such crises [3]. To reduce the morbidity and mortality associated with these overwhelming situations, measures of disaster medicine were developed to evaluate the knowledge, attitude, and preparedness of HCPs [4].

The Eastern Mediterranean Region (EMR) remains the leading theater for the world’s largest humanitarian emergencies [5]. Located in the heart of the EMR, Lebanon is highly disposed to conflicts and man-made disasters. Since the mid-1970s, Lebanon has been in a continuous state of instability due to disabling disasters, starting from a 16-year civil war, passing by several recurrent governmental changes and failures, thus reaching the current disabling economic crisis [6]. This situation has been aggravated since the early 2010s because of the massive influx of Syrian refugees into Lebanon. According to the United Nations High Commissioner for Refugees (UNHCR), Lebanon is now home to approximately 1.5 million refugees (2023) [7]. Additionally, Lebanon has been faced with numerous healthcare emergencies, from the COVID-19 pandemic to the aftermath of one of the largest non-nuclear explosions, the “Beirut explosion”, in August 2020, resulting in countless casualties and displacements [8]. Recent conflicts in the south of the country have exacerbated the nation’s challenges. Until March 2024, reports from the Ministry of Public Health (MoPH) depict a grim reality: 914 injuries and 195 fatalities due to ongoing armed clashes, including at least 42 civilian casualties. Moreover, 90,000 Lebanese citizens are displaced, seeking refuge in approximately 300 makeshift shelters, highlighting the severe strain on Lebanon’s resources and healthcare system [9].

The readiness of Lebanon’s healthcare system to manage disasters has been severely compromised, as it struggles with poor infrastructure and the added burdens stemming from the 2019 economic collapse and the ongoing Syrian refugee crisis [10]. To date and to the best of our knowledge, few studies have evaluated the disaster preparedness of healthcare professionals in the EMR. Therefore, recognizing Lebanon’s geographic vulnerability to disasters, this study aims to evaluate the knowledge, attitude, and readiness to practice associated with disaster medicine preparedness among healthcare professionals in Lebanon. It seeks to identify strengths and weaknesses, focusing on improving programs and readiness strategies to make the healthcare system better prepared to respond to emergencies.

## 2. Methods

### 2.1. Study Design

A cross-sectional observational study was conducted to gain more insight into healthcare professionals’ disaster medicine preparedness during the Beirut blast and the COVID-19 pandemic. The study was completed at the Lebanese American University Medical Center—Rizk Hospital (LAUMCRH), one of the main private university hospitals in Beirut, Lebanon. The study was approved by the Lebanese American University Institutional Review Board (IRB). Approval Code: IRB #: LAUMCRH.RS1.10/Jan/2022. Data were collected between January and March of 2022.

### 2.2. Study Participants

Three groups participated in this cross-sectional epidemiological study: physicians (faculty members and residents), medical students in clinical years, and postdoctoral research scholars. Healthcare professionals, including practicing physicians and residents, play a crucial role in disaster management by providing immediate medical care and coordinating response efforts. Medical students in their clinical years assist with patient care and help maintain hospital operations. Postdoctoral research scholars, as graduate medical doctors, bring advanced training to disaster responses, contributing their expertise in clinical practice, research, and leadership. Including all of the above-targeted professionals, which is essential for disaster scenarios, allows a comprehensive and effective utilization of all available resources.

The inclusion criteria were as follows: health professionals (including practicing physicians and residents) affiliated with LAUMCRH; medical students in their clinical years (third year and above) at the Lebanese American University; postdoctoral research scholars working at LAUMCRH during the study period. The exclusion criteria included individuals who were not affiliated with LAUMCRH, including health professionals and medical students in their first and second preclinical years.

### 2.3. Data Collection

An online questionnaire survey was conducted to self-report and collect data from participants at LAUMCRH. The population sample consisted of 362 healthcare professionals at our institution, from whom we received 195 responses (54% response rate). Using Cochran’s equation, we calculated the required sample size to be 187, considering a 5% margin of error, a 95% confidence level, and an estimated proportion of 50% for variability within the population. To achieve the desired sample size, we employed strategies such as sending multiple reminders at regular intervals to encourage survey completion with in-hospital word-spreading about the study to encourage participation.

### 2.4. Knowledge, Attitude, and Readiness to Practice

A standardized and validated questionnaire was used to examine the knowledge, attitude, and readiness to practice (KArP) among participants [4]. In brief, knowledge was defined as being knowledgeable about disaster readiness with acquaintanceship obtained through practical experience. The knowledge section included 22 close-ended binary questions (yes/no) with scores ranging from 0–22. Participants’ knowledge scores were divided into three categories according to points: low (<7 points; 25th quartile), moderate (7–12 points; 25th–75th quartiles), and high scores (>12 points; 75th quartile).

Attitude was defined as a settled way of acting, affected by experience or thoughts about the disaster. The attitude section included 16 Likert scale questions with scores ranging from 16 to 80. Participants with a score of less than 42 were considered to have a low attitude score (25th quartile), moderate attitude score if they had a score between 42 and 56 (25th–75th quartiles), and high if they had greater than 56 points (75th quartile).

Readiness to practice was designed as a condition of training and motivation to exercise if a disaster takes place. It consisted of 11 Likert scale questions with scores ranging from 11 to 55 points. Participants with scores lower than 31 points (25th quartile), those with scores between 31 and 38 points (25th–75th quartiles), and those with scores greater than 38 points (75th quartile) were considered as low, moderate, and high readiness to practice scores, respectively.

### 2.5. Data Analysis

The socio-demographic characteristics of the participants were reported as a frequency (%) for the categorical variables and a mean (SD) for the continuous variables. We examined the association between the participants’ socio-demographic characteristics and knowledge, attitude, readiness to practice, and total KArP scores. The Kruskal–Wallis test was used to examine the differences in gender and knowledge, attitude, readiness to practice, and total KArP scores.

The Kruskal–Wallis test was used because the data were not normally distributed. We used Pearson’s correlation test to examine the correlation between knowledge, attitude, and readiness to practice. The correlation coefficient (r) was used to measure the strength and direction of the relationship among the different variables, ranging from −1 to +1. A coefficient of 0 means no relationship, +1 signifies a positive correlation, and −1 indicates a negative correlation [11]. All statistical analyses were completed using STATA Version 16.

## 3. Results

A total of 195 participants were included in the study. The average age of the participants was 31 years old (±11.4), and 52% (*n* = 101) were men. Participants’ levels of education were distributed among the different academic levels: 26% were medical students in their clinical years, 18.5% were PGY-1, 12.3% were PGY-2, 9.2% were PGY-3, 7.7% were PGY-4, 4.1% were PGY-5, 1.0% were PGY-6, 3.6% were postdoctoral research scholars, and 18% were practicing physicians (Table 1).

**Figure 1:**
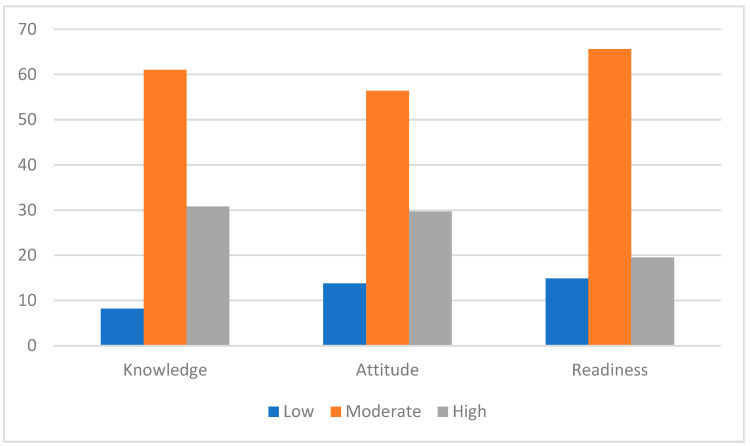
Percentage distribution of knowledge, attitude, and readiness levels across participants.

Figure 1 depicts the distribution of participants’ levels in the different preparedness scores as percentages. In the knowledge section, 16 (8.2%) participants had low knowledge scores, 119 (61.0%) participants had moderate scores, and 60 (30.8%) participants had high knowledge scores. In the attitude section, 27 (13.9%) participants had low scores, 110 (56.4%) had moderate, and 58 (29.7%) participants had high attitude scores. In the readiness to practice section, 29 (14.9%) participants had low scores, 128 (65.6%) had moderate, and 38 (19.5%) participants had high readiness to practice scores (Figure 1).

Participants’ ages were positively correlated with the readiness to practice and KArP scores, and older participants reported a better readiness to practice and better KArP scores. A significant difference was observed in the knowledge, attitude, readiness to practice, and total KArP scores according to gender, with men scoring significantly higher scores in all those categories compared to women (Table 2).

A significant difference was found between the level of education and readiness to practice scores. Participants in their sixth year of postgraduate training (PGY-6) demonstrated higher levels of readiness to practice, with a score of 41, compared to medical students, whose scores ranged from 32.5 to 34.9 (Table 2). However, no significant difference was observed between the level of education and knowledge, attitude, and total KArP scores. This suggests that participants’ levels of education did not impact their scores in these areas, regardless of the years of experience or exposure in the medical field (Table 2).

In Table 3, the knowledge scores were positively correlated with the attitude, readiness to practice, and overall KArP scores (r = 0.53, r = 0.45, r = 0.69, respectively). This means that as the knowledge scores increase, the attitude, readiness to practice, and overall KArP scores also tend to increase. The attitude scores were positively correlated with the readiness to practice scores and strongly positively correlated with the total KArP scores (r = 0.67, r = 0.94, respectively). This implies that as the attitude scores increase, the readiness to practice scores and total KArP scores also tend to increase. The readiness to practice scores were strongly positively correlated with the KArP scores (r = 0.84), indicating that as the readiness to practice scores increase, the KArP scores also tend to increase.

## 4. Discussion

In our study, Lebanese HCPs’ knowledge, attitude, and readiness to practice in disasters were evaluated. The majority of the participants had moderate scores for all three categories. These findings highlight the need for further research on the matter to determine the means necessary for improving preparedness for the HCPs in disastrous circumstances. As such, it is essential to take the correct initiatives to improve disaster KArP in Lebanon, a place where disasters tend to occur more frequently.

According to recent statistics, the EMR is more vulnerable to disasters due to unexpected political and social instabilities, accounting for about a quarter of global mass casualty incidents (MCI), and is leading in terrorist events [12,13]. The influx of refugees has further strained Lebanon’s already unstable emergency healthcare system, limiting its capacity to handle daily and global crises [14]. The COVID-19 pandemic exacerbated this, with 80% of the healthcare budget allocated to acute emergencies, leaving hospitals unprepared for unforeseen catastrophes [15].

In August 2020, a massive explosion occurred in the Beirut Port, leaving behind 220 victims, more than 6500 injured, and more than 300,000 homeless people [16]. This event posed significant challenges for Lebanese medical professionals, with major hospitals becoming non-functional or overwhelmed [17]. Although some of the Lebanese medical teams were prepared for such a disaster, most of them were not, which made it difficult to manage this massive catastrophe in such debilitating circumstances of a failing, nearly broken state [18]. Since October 2023, armed conflicts along Lebanon’s southern border have caused extensive loss of life, displacement of over 91,000 people, and significant hardships, highlighting the critical need for well-defined crisis management strategies [19]. This underscores the importance of enhancing preparedness and readiness among healthcare professionals to handle various disasters.

The eleventh sustainable development goal (SDG 11), announced by the United Nations, aims to make cities inclusive, safe, resilient, and sustainable [20,21]. This includes improving disaster resilience and resource efficiency and reducing the impact of disasters. Disaster risk reduction (DRR) is essential, as it promotes education and healthy lives [22]. Our study focuses on evaluating the preparedness of the Lebanese healthcare system, identifying areas for improvement, and helping bridge gaps to achieve safer and more sustainable cities in line with SDG 11.

### 4.1. Knowledge of Disaster Medicine

The results of HCPs’ knowledge regarding disaster medicine revealed that only 30.8% of the respondents had high knowledge scores, while most respondents had moderate scores. No significant correlations were found with age or level of education. However, gender emerged as a significant variable, with men having significantly higher knowledge scores than women. This gender trend differs from the findings of a Qatari study, which reported no significant gender-based differences [4], and a study conducted in the UAE that also found no statistically significant differences between males and females in knowledge scores [23]. This variation may reflect the differences in educational opportunities, professional experiences, or societal roles across different regions and populations. However, unlike the study in Qatar and the UAE, with a skewed gender distribution, our sample had a more balanced representation, which may offer a more accurate picture of knowledge levels. This discrepancy underlines the importance of equitable training opportunities and highlights the need for integrating disaster medicine education into medical curricula. Ashcroft et al.’s literature reviewed this by demonstrating the positive impact of disaster training programs on medical students, improving their knowledge, skills, and attitudes [24]. Additionally, symposiums and workshops can further enhance knowledge among all HCPs at all levels [25].

### 4.2. Attitude Regarding Disasters

This study also showed that the majority of respondents (56.4%) had a moderate attitude. Yet again, only gender was found to be a significant variable, with men having higher scores for attitude than women, which is consistent with the findings from a cross-sectional study conducted in Yemen involving 1093 HCPs. The research indicated that attitude and willingness to respond were higher among male healthcare professionals [26]. Another study, which was conducted by Naser et al., included 531 health professionals and found that the participants overwhelmingly expressed the importance of having well-defined emergency plans in their facilities, managed by a proactive disaster committee responsible for effective plan implementation. Moreover, a significant 82.9% believed that conducting drills, workshops, or other simulation exercises in their workplaces was essential, recognizing them as valuable components of disaster training [27]. These findings highlight the importance of having well-defined emergency plans, as they ensure structured responses and are complemented by increased workshops and drills. Such initiatives are crucial in ensuring that healthcare workers feel adequately prepared and confident in their response capabilities, ultimately leading to more effective emergency management.

### 4.3. Readiness to Practice in Times of Disaster

In terms of readiness to practice, our study reveals that 65.6% of participants exhibited moderate scores, with 14.9% and 19.5% reporting low and high readiness, respectively, indicating a varied preparedness level among HCPs. Interestingly, both age and educational level emerged as significant factors affecting readiness for practice. Respondents with higher educational qualifications, such as postgraduate year (PGY-6), demonstrated residents with a greater readiness for practice compared to medical students. Interestingly, older participants also exhibited a positive correlation between age and readiness for practice, likely due to their increased experience and additional training opportunities over time.

Our findings revealed a gender-related disparity in attitudes towards disaster preparedness, with men reporting higher readiness scores than women. This is consistent with prior research, highlighting diverse levels of readiness among HCPs in a disaster management context [26]. Our study also explored the correlation between the different variables, revealing positive associations between the knowledge scores, readiness, attitude, and overall KArP scores. The participants who were more prepared had better overall scores. These significant results show the importance of preparing all HCPs comprehensively for disaster situations. Self-efficacy, which represents the confidence of HCPs in their ability to respond effectively to public health crises, has been identified as a crucial factor associated with a willingness to participate in disaster response efforts [28]. Therefore, interventions aimed at improving self-efficacy through tailored training programs in disaster preparedness may serve to enhance the willingness of healthcare workers to engage actively in disaster management [26]. Given the broad scope of disaster medicine, encompassing care for victims of both natural and human-made disasters, including disaster management, it is imperative for healthcare workers to possess a willingness to respond effectively.

Our findings suggest potential interventions to enhance disaster preparedness among HCPs, such as integrating disaster medicine courses into medical school and residency programs in Lebanon. Other studies have similarly advocated for the inclusion of disaster medicine programs and skill training in curricula, highlighting the importance of proactive measures to improve readiness and crisis management skills among emerging HCPs [29,30]. Implementing such strategies may play a pivotal role in shaping the attitudes of younger generations of HCPs, fostering better preparedness, and ultimately enhancing their ability to manage unforeseen disasters effectively within their communities.

### 4.4. Strengths and Limitations

This study has several strengths. The participants included in this study are enrolled in one of the largest university hospitals in Lebanon and have diverse educational and clinical backgrounds. Furthermore, the hospital’s direct involvement in managing victims of the Beirut blast on 4 August 2020 highlights how participants have personally engaged in disaster response efforts. A standardized, validated tool was used to assess knowledge, attitudes, and readiness to practice, which enhanced the generalizability of our findings. In addition, we examined these findings among medical professionals after the largest non-nuclear blast in history [31]. This study also has some limitations that must be kept in mind when interpreting our study findings. The participants in this study were comprised of Lebanese nationals and were Middle Eastern, which limits the generalizability of our findings to other ethnic/racial groups. Second, only health professionals were included in this study, comprising medical students, residents, postdoctoral fellows, and practicing physicians. Paramedical professionals, such as nurses, pharmacists, and other healthcare professionals, were not included. Moreover, the small sample size of the PGY-6 subgroup is a limitation, suggesting that the results may be over-interpreted. Finally, our study findings depend on self-reported data from a survey, which may have caused biases.

## 5. Conclusions

Our study showed that healthcare professionals in an academic institution in Lebanon had moderate scores in knowledge, attitude, and readiness to practice regarding disaster management. Older participants and those with a higher level of education reported better readiness to practice. Men, compared to women, had significantly higher scores in all categories. Further studies should explore these associations in a larger cohort of HCPs with more detailed characteristics to identify and target the barriers to KArP. Disaster preparedness, through training and implementation of reformed, strong, and strategic curricula at the institutional levels, may improve outcomes in future disaster encounters.

## Figures and Tables

**Table 1:** Socio-demographic characteristics of participants.

Socio-Demographic Characteristics	Frequency (*n* = 195)	Percentage (%)
Age, mean, years (SD)	30.6 (11.4)	
Gender		
Female	94	48.2
Male	101	51.8
Level of Education		
MS-3	26	13.3
MS-4	24	12.3
PGY-1	36	18.5
PGY-2	24	12.3
PGY-3	18	9.2
PGY-4	15	7.7
PGY-5	8	4.1
PGY-6	2	1.0
Postdoctoral Research Scholar	7	3.6
Practicing Physicians	35	18.0

Abbreviations: SD = standard deviation; MS = medical student; PGY = postgraduate year.

**Table 2:** Socio-demographic and academic characteristics according to attitude, knowledge, and readiness to practice scores.

Socio-Demographic Characteristics	Knowledge Score (n = 195)	*p*-Value	Attitude Score (n = 195)	*p*-Value	Readiness to Practice Score (n = 195)	*p*-Value	KArP (Total Score)	*p*-Value
Age (years)	r = 0.23	0.84	r = 0.11	0.43	r = 0.02	**<0.01**	r = 0.13	**0.02**
Gender								
Female	10.3 (3.1)	**0.01**	49.5 (8.8)	**<0.01**	33.9 (5.0)	**<0.01**	93.7 (14.0)	**<0.01**
Male	11.5 (3.7)		54.2 (9.5)		36.1 (5.5)		101.9 (1.6)	
Level of Education								
MS-3	10.5 (3.5)	0.31 *	50.3 (9.5)	0.13 *	34.9 (5.7)	**0.04** *	95.7 (16.1)	0.07 *
MS-4	10.5 (3.0)		47.8 (8.6)		32.5 (5.6)		90.8 (14.1)	
PGY-1	10.5 (3.3)		51.3 (11.0)		35.3 (6.0)		97.1 (18.3)	
PGY-2	10.8 (3.5)		52.1 (7.9)		36.4 (4.5)		99.3 (13.7)	
PGY-3	11.2 (3.8)		55.9 (7.6)		36.2 (4.7)		103.2 (12.9)	
PGY-4	11.6 (2.7)		53.2 (6.8)		35.8 (3.8)		100.6 (10.1)	
PGY-5	10.6 (3.7)		53.4 (6.2)		35.1 (2.9)		99.1 (11.7)	
PGY-6	12.5 (2.1)		58.5 (10.6)		41 (5.7)		112 (18.4)	
Postdoctoral Research Scholar	9.0 (2.4)		48.3 (6.0)		31.8 (2.0)		89.1 (6.9)	
Practicing Physicians	12.3 (3.8)		54.2 (11.0)		34.7 (6.2)		101.2 (18.5)	

Abbreviations: r = correlation coefficient; n = sample size; SD = standard deviation; MS = medical student; PGY = postgraduate year. Note: * = Kruskal–Wallis test. Bold *p*-value = significant.

**Table 3:** Association between knowledge, attitude, and readiness to practice scores.

	Statistical Values	Attitude	Readiness to Practice	KarP
Knowledge	r	0.53	0.45	0.69
	*p*-value	0.00	0.00	0.00
	n	195	195	195
Attitude	r	-	0.67	0.94
	*p*-value	-	0.00	0.00
	n	-	195	195
Readiness to Practice	r	-	-	0.84
	*p*-value	-	-	0.00
	n	-	-	195

Abbreviations: r = correlation coefficient; n = sample size.

## Data Availability

The data presented in this study are available on request from the corresponding author due to restrictions related to confidentiality agreements and privacy concerns.
